# Impact of COVID-19 Lockdown Measures on the Incidence of Peritonsillar Abscess: A Retrospective Study

**DOI:** 10.3390/life14121554

**Published:** 2024-11-26

**Authors:** Peter Kiss, Ulrich Moser, Michael Habenbacher, Katharina Walla, Jakob Pock, Alexandros Andrianakis

**Affiliations:** Department of Otorhinolaryngology, Medical University of Graz, 8010 Graz, Austria

**Keywords:** SARS-CoV-2, pandemic, lockdown, infectious disease, public health

## Abstract

Background/Objectives: During the COVID-19 pandemic, Austria, like many European countries, implemented lockdown measures to curb viral transmission. These public health interventions, including social distancing and improved hygiene, were anticipated to affect various infectious diseases. This study aimed to assess whether the incidence of peritonsillar abscess, a severe upper respiratory inflammatory disease, decreased during the lockdown period of 2020 compared to previous years. Methods: This retrospective study analyzed all patients diagnosed with peritonsillar abscess from 2010 to 2020 at the Department of Otorhinolaryngology, Medical University of Graz. Patients were grouped based on the onset of disease into two periods: the lockdown period (16 March–29 May) and the rest of the year = Period 2 (1 January–15 March and 30 May–31 December). The incidence during the lockdown period of 2020 was compared to the incidence in the same timeframe in the previous years from 2010 to 2019 using chi-squared tests and Poisson regression models. Results: A total of 1768 patients (female: 44%, male: 56%; mean age: 34.6 ± 18.4 years) diagnosed with peritonsillar abscess were treated between 2010 and 2020 at the Department of Otolaryngology, Medical University of Graz. The analysis revealed that the proportion of peritonsillar abscesses in Period 1 in 2020 (15/127, 12%) was significantly lower than the mean proportion in Period 1 from 2010 to 2019 (36/164, 22%) (*p* = 0.004). The incidence rate of peritonsillar abscess cases per year in Period 1 (16 March–29 May) was significantly higher in each previous year from 2010 to 2019 compared to 2020 (*p* < 0.05). Conclusions: The findings suggest that the COVID-19 lockdown measures contributed to a reduction in the incidence of peritonsillar abscess, highlighting the broader impact of public health interventions on infectious diseases. Further research is needed to explore the effects on other respiratory infections and diseases.

## 1. Introduction

In December 2019, Severe Acute Respiratory Syndrome Coronavirus 2 (SARS-CoV-2) was identified in Wuhan, Hubei Province, China [1]. The novel Coronavirus caused Coronavirus disease 2019 (COVID-19), a global health emergency that prompted governments worldwide to implement stringent public health measures. Austria, like many European countries, applied lockdown measures between March and May 2020 to limit viral transmission [2,3].

Lockdowns are exceptional public health measures aimed at containing the pandemic. While their effectiveness and advantages are being discussed, some authors underlined the existence of collateral effects, such as the abrupt reduction or delay in emergency department admissions during the COVID-19 outbreak [4,5,6]. In a recent expression of concern, Feral-Pierssens et al. [7] provided two main reasons for the reduction in emergency visits including that patients may have adopted alternative strategies to seek medical help, such as teleconsultation and COVID-free walk-in clinics. Alternatively, patients may have actively avoided or even fled emergency departments due to the concern of being infected with SARS-CoV-2 in the hospital. In specific cases, the decline in emergency room visits may at least in part be explained by the closures themselves, which induced social stillness and improved awareness of hygienic habits. This may have led to a decreasing number of traumatic events and infections of the upper respiratory tract, for example. In contrast, there is no theoretical reason to assume that the preventive measures against COVID-19 induced a reduction in the incidence of medical conditions such as stroke, myocardial infarct or acute abdominal problems. The apparent reduction in patients with these conditions may actually be due to the fact that individuals chose not to seek medical attention, maybe because the symptoms of COVID-19 are similar to acute coronary syndromes, for instance, or more simply because of fear of the infection itself.

Due to the drastic increase in the number of COVID-19 cases in Austria and in the neighboring country Italy, the Austrian government enacted the first COVID-19-related lockdown on 16 March 2020 [8]. The gradual reopening started in May 2020. During the initial—strictest—phase, nurseries, kindergartens, schools, non-essential shops/businesses, restaurants and other gastronomic facilities were closed. Similarly, to other regions, also the university hospitals adapted to the situation by adopting measures aimed at reinforcing the reserves of healthcare systems that were expected to collapse due to the pandemic. Our Department of Otolaryngology is an academic tertiary care center. The outpatient care is almost entirely focused on acute, emergency care—while, of course, retaining the possibility of elective visits as much as possible. In inpatient care, priority was set to the continuation of non-emergency therapies, which could not be postponed without significant risks or foreseen damage, and to the uninterrupted provision of care for our oncology patients.

During the lockdown period, the number of personal contacts dropped drastically, and basic hygiene rules were followed to a much higher degree. Recent studies reported a significant decrease in incidence of upper airway infections during the lockdown period [9,10,11,12]. However, as most upper respiratory infections are uncomplicated and do not require immediate professional medical help, this decrease might be confounded by avoiding or delaying care out of fear of contracting COVID-19. Among upper airway inflammatory conditions that require immediate professional medical help, the peritonsillar abscess is the most common disease with an annual incidence between 30 and 37 cases per 100,000 individuals [13,14]. Peritonsillar abscess is a localized inflammation in which pus accumulates between the fibrous capsule of the tonsil and the superior pharyngeal muscle. It is a pathology with a fulminant course and presents with severe symptoms (malaise, fever, progressively severe sore throat, dysphagia and the typical “hot potato” voice) that increase over time, so that a patient inevitably needs to seek medical attention. Furthermore, the treatment of this disease takes place under standardized conditions by an otolaryngologist. At our clinic, this disease is treated in inpatient care without exception, regardless of the chosen method of treatment. These characteristics, namely a sufficiently high incidence, a severe course requiring medical attention and the need for hospitalization, make the peritonsillar abscess an ideal representative candidate to investigate the impact of the COVID-19 lockdown on the reduction in upper respiratory tract infections. To the best of our knowledge, no such study has yet investigated this specific research question.

Therefore, the current study aimed to evaluate whether the incidence of peritonsillar abscess decreased in the lockdown period of 2020 compared to the previous years. We hypothesized that the lockdown measures induced a relevant reduction in the incidence of peritonsillar abscesses.

## 2. Materials and Methods

### 2.1. Subjects

This retrospective study involved all patients diagnosed with peritonsillar abscess between 1 January 2010 and 31 December 2020 at the Department of Otolaryngology, Medical University of Graz. Inclusion criteria were: (1) diagnosis of peritonsillar abscess confirmed based on the presence of pus during needle aspiration/incision, in conjunction with specific clinical signs and symptoms like unilateral tonsillar swelling, deviation of the uvula, severe sore throat, odynophagia, trismus, and the characteristic ‘hot potato’ voice. In cases with diagnostic uncertainty, computed tomography was utilized to confirm the diagnosis; (2) inpatient treatment. Exclusion criteria were differential diagnosis to peritonsillar abscess including peritonsillar cellulitis, dental infections, neoplasia, angioedema, internal carotid artery aneurysm and Vincent’s tonsilitis. At our department, a standardized clinical protocol for the management of peritonsillar abscess is applied. All patients are hospitalized for treatment. The primary therapy is incisional drainage under local anesthesia. In the case of retrotonsillar abscess, unsuccessful incision drainage, complicated peritonsillar abscess or lack of patient compliance, an abscess tonsillectomy is performed. All patients receive intravenous antibiotic therapy with amoxicillin/clavulanic acid (or clindamycin in case of penicillin allergy) and metronidazole and analgetic therapy.

### 2.2. Study Design

For each year of the study period (2010–2020), patients were stratified according to their onset of peritonsillar abscess into two time periods: Period 1: Disease onset between 16 March and 29 May; which corresponds the COVID-19 lockdown time period in 2020 in Austria; and Period 2: Disease onset between 1 January and 15 March and 30 May–31 December, representing the remaining part of the year. The total number of patients and demographics including age and sex were descriptively analyzed for each year of the study duration and for both specified time periods of each year.

We compared the proportion of cases that occurred in Period 1/Period 2 in 2020 to the mean proportion of cases in the same two time periods from 2010 until 2019. Our hypothesis was that the amount of peritonsillar abscess cases in the lockdown period (Period 1) in 2020 was significantly lower than in the previous years, while the proportion of cases in Period 2 remained unchanged.

### 2.3. Statistical Analysis

Continuous variables are reported as mean and standard deviation, categorical variables as absolute and relative frequencies. A chi-squared test was performed to compare the proportion of peritonsillar abscess cases in the lockdown period in 2020 against the mean proportion of cases in the same time period of the previous years from 2010 to 2019, as well as for each year separately. Additionally, we performed two Poisson regression models predicting the number of peritonsillar abscess cases in Period 1 and 2, respectively, as a function of year. We included an offset in the models to account for the yearly variation in the number of patients seen in our department. Year was included in the analysis as a factor, with 2020 as reference level. This strategy allowed us to test for differences in the rate of the diagnoses between 2020 and each previous year. Mean and variance of the dependent variable were inspected for each model in order to check the main assumption that mean = variance. In case this assumption was not met, we planned to apply a quasi-Poisson model that could account for over dispersion. Unfortunately, the quasi-Poisson model did not converge when applied to the models with year as a factor. We thus performed a sensitivity analysis by computing a model with year as a continuous predictor and a dummy variable, which was 1 for 2020 and zero for the other years. This way we could specifically test whether 2020 was significantly related to a change in the rate of the diagnosis compared to the previous years. A *p* value of ≤ 0.05 was considered significant. All statistical analyses were conducted using R version 4.1.0.

### 2.4. Ethical Considerations

The study was approved by the Institutional Review Board (IRB) (approval code: 33-096 ex 20/21, approval date: 17 November 2020). The requirement of written informed consent from the patients was waived from the IRB due to the retrospective nature of the study. The study was performed in accordance with the ethical guidelines of the Declaration of Helsinki.

## 3. Results

**Figure 1:**
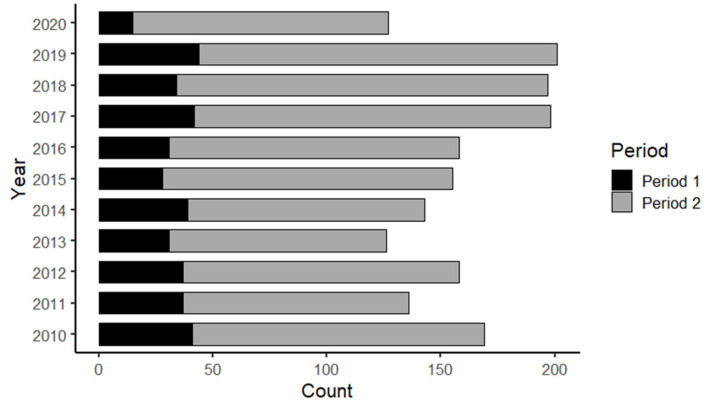
The plot shows the absolute number of peritonsillar abscess cases in Period 1: 16 March–29 May = Lockdown (black) and Period 2: 1 January–15 March and 30 May–31 December (gray) in each year.

A total of 1768 patients diagnosed with peritonsillar abscess were treated between 1 January 2010, and 31 December 2020, at the Department of Otolaryngology, Medical University of Graz. Figure 1 shows the absolute number of peritonsillar abscess cases for both, Period 1: 16 March–29 May = Lockdown (black) and Period 2: 1 January–15 March and 30 May–31 December (gray) in each year from 2010 to 2020. The proportion of peritonsillar abscesses in Period 1 in 2020 (15/127, 12%) was statistically significantly lower than the mean proportion in Period 1 in 2010–2019 (36/164, 22%) (*p* = 0.004).

Table 1 shows the absolute and relative frequency of cases in both Period 1: 16 March–29 May = Lockdown; and Period 2: 1 January–15 March and 30 May–31 December per study year from 2010 to 2020 together with demographics including age and sex.

The results of the Poisson regression models are reported in Table 2. The incidence rate of peritonsillar abscess cases per year in Period 1 (16 March–29 May) was significantly higher in each previous year from 2010 to 2019 compared to 2020 (*p* < 0.05). In contrast, there was no significant difference in the incidence rate of peritonsillar abscess cases in 2020 compared to previous years, concerning Period 2 (1 January–15 March and 30 May–31 December). Note that data indicated over dispersion (mean < variance) in both periods of interest. We thus performed a sensitivity analysis comparing the Poisson with a quasi-Poisson regression model. The results of these analyses were very consistent in both periods of interest. In Period 1, the year 2020 was related to a significant decrease in the number of visits as compared to the previous years (estimate = −0.66, SEPoisson = 0.28, pPoisson = 0.018, SEquasi-Poisson = 0.24, pquasi-Poisson = 0.024). In Period 2, there was no significant difference in the rate of the diagnosis in 2020 compared to the previous years, (estimate = −0.13, SEPoisson = 0.11, pPoisson = 0.223, SEquasi-Poisson = 0.14, pquasi-Poisson = 0.350).

## 4. Discussion

The present study investigated whether the lockdown measures established in March 2020 in Austria to contain the spread of the COVID-19 pandemic induced a relevant reduction in the number of peritonsillar abscesses compared to the previous years. Our hypothesis was driven by the assumption that the observance of social distancing rules and hygienic measures induced a relevant decrease in the transmission of pathogens, which may have in turn affected the incidence of a benchmark inflammatory disease of the upper respiratory tract, the peritonsillar abscess. We specifically focused on this disease because it can hardly be undetected: it has a sufficiently high incidence of 30–37/100,000 per year [13,14], a severe course that needs medical attention and its treatment requires hospitalization. In line with our hypothesis, we observed a significantly lower incidence of peritonsillar abscesses during the COVID-19 lockdown phase (March–May) in 2020 compared to the same period in the previous years. Note that we took into account the annual variation in visits in the department as offset in our analysis and therefore the findings reflect the reduction in the incidence of the benchmark disease over and above changes in the annual number of visits.

The COVID-19 pandemic, and the lockdowns that ensued, dramatically reshaped healthcare delivery and the epidemiology of many diseases, including upper respiratory tract infections. As the virus spread across the globe, countries imposed strict public health measures to curb transmission, including social distancing, strict hygiene rules, mask-wearing, and lockdowns, which led to both anticipated and unintended consequences across various sectors of healthcare [15,16]. Our study adds to the growing body of literature that examines the collateral effects of these measures, specifically focusing on the incidence of peritonsillar abscess during the lockdown period.

Several studies have documented the impact of the COVID-19 pandemic lockdown on various health conditions, including but not limited: mental health, nutritional status and physical activity, cancer outcome, inflammatory diseases [15,16,17,18,19,20,21]. Furthermore, the lockdown led to significant changes in daily life like reduced social interactions, improvements in personal hygiene, and decreased pollution, all of which contribute to a lower transmission rate of infectious diseases. However, lockdowns also posed challenges for healthcare access, with some individuals avoiding or delaying care out of fear of contracting COVID-19 in healthcare settings [15,16]. These factors combined to create a distinct epidemiological shift in the incidence of upper respiratory infections during the pandemic.

Several studies from various regions have documented a decline in upper respiratory infections during the COVID-19 lockdown [9,10,11,12]. For example, Haapanen et al. [9] observed a 51% reduction in ENT infections during the 2020 lockdown compared to previous years. Similarly, Kelloniemi et al. [10] noted an abrupt cessation of RSV and influenza infections during the pandemic in Finland. This sharp decline in respiratory infections can be attributed to the implementation of stringent public health measures such as social distancing, mask mandates, and stay-at-home orders, all of which significantly curbed the spread of pathogens responsible for respiratory infections. These findings support the hypothesis that lockdown measures contributed to an overall decrease in infectious diseases. However, as most upper respiratory infections are uncomplicated and do not require immediate professional medical help, this decrease might be confounded by avoiding or delaying care out of fear of contracting COVID-19. In our study, we therefore focused on the inflammatory condition peritonsillar abscess, which shows a severe clinical course that needs immediate medical attention, and its treatment requires hospitalization. In this context, we observed a significant reduction in the number of peritonsillar abscess cases during the 2020 lockdown, consistent with global trends reported in similar studies. This reduction likely reflects the effects of social distancing, mask-wearing, and improved hygiene practices during the pandemic.

While our findings align with broader research on the impacts of the COVID-19 lockdown, our study provides particularly valuable insights into the specific epidemiology of peritonsillar abscess during this period. A recent study by Cidlinsky et al. [22] investigated the effects of the pandemic on the frequency and therapeutic management of peritonsillar abscess cases in Germany and noted that while the number of cases decreased, there was no significant change in the percentage of peritonsillar abscess among ENT emergencies. Their approach differed significantly in terms of scope and timeframe compared to our study. The focus of their study was on the changes in treatment practices and healthcare capacity, rather than on the epidemiological trends we emphasize. They examined cases over a two-year period following the initiation of the lockdown, which included a substantial period without lockdown restrictions, and compared them to cases from the two years before the pandemic. This method, while comprehensive, included long stretches where public health measures had been relaxed, and societal behavior had returned towards pre-pandemic norms. As a result, the broader comparison may have diluted the specific impact of the strict lockdown on peritonsillar abscess incidence by including data from both restricted and unrestricted periods. In contrast, our study narrowed its focus exclusively to the lockdown itself, comparing the incidence peritonsillar abscess during this defined period directly to the equivalent timeframes in the prior years. This approach allowed us to isolate the direct influence of the lockdown, providing clearer evidence that the reduction in cases was closely linked to the public health interventions implemented during this specific time. Moreover, our study evaluated data from a decade, providing a more comprehensive perspective on how public health interventions impact disease incidence in the longer term.

The significant reduction in peritonsillar abscess incidence observed during the 2020 lockdown aligns with broader evidence of the effectiveness of non-pharmaceutical interventions during the pandemic. Studies from the International COVID-19 Research Network have demonstrated that non-pharmaceutical interventions such as social distancing, mask-wearing, and hygiene promotion significantly reduced the transmission of respiratory infections, including COVID-19. By limiting interpersonal interactions and promoting public health awareness, non-pharmaceutical interventions disrupted transmission pathways for various pathogens, leading to declines in diseases like peritonsillar abscess. This finding supports the hypothesis that lockdown measures can mitigate not only viral diseases but also bacterial infections that arise as complications of viral upper respiratory tract infections. The broader implications of these results underscore the importance of integrating lessons from the pandemic into public health strategies. For example, targeted non-pharmaceutical interventions during respiratory disease outbreaks could be employed to reduce healthcare burdens and protect vulnerable populations. However, the long-term feasibility and indirect consequences of such measures, including their social and economic impacts, warrant further investigation. Future studies should also explore the effectiveness of different non-pharmaceutical interventions across diverse populations and healthcare systems to inform evidence-based public health policies [23,24].

We are aware that it is difficult to make a general statement based on a single pathology; however, peritonsillar abscess serves as an excellent benchmark disease due to its acute course, measurable incidence, and severe symptoms. It might be worthwhile and interesting to examine the changes in the number of other infections that require immediate inpatient care such as epiglottic abscess, deep neck abscess and orbital abscess. However, those pathologies do not have a sufficiently high incidence for suitable statistical analysis.

While the findings of this study are compelling, several limitations warrant discussion. First, the retrospective nature of the study introduces potential biases, as we rely on existing data without the ability to control certain confounding factors. In this context, we were unable to provide information on patient comorbidities or routine medication use. These factors could influence susceptibility to infections or healthcare-seeking behavior during the lockdown and should be explored in future investigations. The lack of these data limits our ability to assess whether underlying health conditions contributed to the observed reduction in peritonsillar abscess cases. Furthermore, the study’s reliance on retrospective data precluded the ability to account for behavioral changes during the pandemic, such as altered healthcare-seeking patterns, socioeconomic factors, or variations in access to medical care. These confounders may have influenced our results and require prospective designs for more accurate assessments. Second, the single-center design limits the generalizability of our findings. While our results are consistent with similar studies from other regions, a multicenter approach would have provided more robust and generalizable data. Third, we did not analyze the severity of cases or treatment outcomes due to the retrospective design and data limitations, which could have provided a deeper understanding of the clinical implications of the observed trends. Future studies should incorporate these elements to provide more robust and comprehensive results. Lastly, the study period’s restriction to a specific lockdown duration might also limit our ability to observe the long-term effects of public health measures.

Despite these limitations, the study makes a meaningful contribution by demonstrating the impact of non-pharmaceutical interventions on the incidence of a benchmark upper respiratory condition. It provides a strong foundation for future research aimed at exploring the long-term effects of pandemic measures, investigating the role of patient comorbidities, and adopting advanced statistical approaches to enhance the reliability and generalizability of findings.

## 5. Conclusions

This study demonstrated a significant reduction in the incidence of peritonsillar abscess during the COVID-19 lockdown period in 2020 compared to previous years. Specifically, the proportion of cases in the lockdown period (12%) was substantially lower than the mean proportion in the same timeframe from 2010 to 2019 (22%). These findings highlight the effectiveness of non-pharmaceutical interventions, such as social distancing and improved hygiene, in reducing the transmission of pathogens responsible for upper respiratory infections. They also emphasize the potential for integrating lessons from the pandemic into public health policies to mitigate the spread of infectious diseases and enhance resilience in healthcare systems. Future multicenter and prospective studies are warranted to validate these findings, assess the role of patient-specific factors, and explore the long-term impacts of such interventions on public health.

## Figures and Tables

**Table 1:** Frequency of the overall number of cases per year and number of cases, mean age (M), with standard deviation (SD) and sex in the two periods of interest (Period 1: 16 March–29 May = Lockdown; Period 2: 1 January–15 March and 30 May–31 December).

Year	Cases Whole Year	Cases Period 1	Age in Period 1	Male in Period 1	Age in Period 2	Male in Period 2
	N	N (%)	M (SD)	N (%)	M (SD)	N (%)
2010	169	41 (24%)	34.4 (18.6)	19 (46.3%)	35.1 (17.5)	74 (57.8%)
2011	136	37 (27%)	30.9 (15.1)	19 (51.4%)	34.7 (19.9)	59 (59.6%)
2012	158	37 (23%)	33.2 (16.7)	22 (59.5%)	35.0 (17.7)	80 (66.1%)
2013	126	31 (25%)	40.5 (19.6)	20 (64.5%)	35.0 (16.4)	57 (60.0%)
2014	143	39 (27%)	32.7 (14.6)	24 (61.5%)	32.9 (15.7)	56 (53.8%)
2015	155	28 (18%)	33.8 (17.8)	17 (60.7%)	33.7 (16.1)	73 (57.5%)
2016	158	31 (20%)	32.5 (18.2)	8 (25.8%)	37.5 (18.6)	65 (51.2%)
2017	198	42 (21%)	26.9 (15.6)	25 (59.5%)	34.9 (16.7)	87 (55.8%)
2018	197	34 (17%)	33.1 (18.3)	17 (50.0%)	34.5 (17.2)	84 (51.5%)
2019	201	44 (22%)	35.3 (21.1)	25 (56.8%)	36.7 (17.9)	88 (56.1%)
2020	127	15 (12%)	31.1 (17.6)	8 (53.3%)	35.6 (18.6)	59 (52.7%)

**Table 2:** Results of the Poisson regression models predict the number of cases in Period 1 and Period 2, respectively, as a function of year. The annual number of visits to the Department was included as offset. The reference level for the categorical predictors was the year 2020. Results are presented as incidence rate ratios (IRR) with 95% CI.

Period 1		95% CI			
	IRR	Lower bound	Upper bound	z	*p*
Year 2010	2.36	1.33	4.39	2.84	<0.01
Year 2011	2.25	1.26	4.23	2.65	0.01
Year 2012	2.30	1.29	4.32	2.72	0.01
Year 2013	1.88	1.03	3.58	2.01	0.04
Year 2014	2.43	1.37	4.54	2.92	<0.01
Year 2015	1.66	0.9	3.19	1.59	0.11
Year 2016	1.81	0.99	3.44	1.88	0.06
Year 2017	2.37	1.34	4.41	2.87	<0.01
Year 2018	1.76	0.98	3.32	1.82	0.07
Year 2019	2.23	1.27	4.13	2.68	0.01
**Period 2**		**95% CI**			
	IRR	Lower bound	Upper bound	z	*p*
Year 2010	0.99	0.76	1.27	−0.12	0.91
Year 2011	0.81	0.61	1.06	−1.56	0.12
Year 2012	1.01	0.78	1.3	0.05	0.96
Year 2013	0.77	0.59	1.01	−1.86	0.06
Year 2014	0.87	0.66	1.13	−1.05	0.29
Year 2015	1.01	0.78	1.3	0.08	0.94
Year 2016	0.99	0.77	1.28	−0.07	0.94
Year 2017	1.18	0.93	1.51	1.33	0.18
Year 2018	1.13	0.89	1.44	0.98	0.33
Year 2019	1.06	0.84	1.36	0.5	0.62

## Data Availability

The original contributions presented in the study are included in the article, further inquiries can be directed to the corresponding author.
